# Deubiquitination in prostate cancer progression: role of USP22

**DOI:** 10.20517/2394-4722.2020.23

**Published:** 2020-06-18

**Authors:** Nivedita Nag, Samikshan Dutta

**Affiliations:** 1 Department of Microbiology, Sister Nibedita Government General Degree College for Girls, Kolkata 700027, India.; 2 Department of Biochemistry and Molecular Biology, University of Nebraska Medical Center, Omaha, NE 68198-5870, USA.

**Keywords:** USP22, prostate cancer, SAGA, Deubiquitin

## Abstract

Prostate cancer (PCa) is the leading cause of cancer death in men. With more therapeutic modalities available, the overall survival in PCa has increased significantly in recent years. Patients with relapses after advanced secondgeneration anti-androgen therapy however, often show poor disease prognosis. This group of patients often die from cancer-related complicacies. Multiple approaches have been taken to understand disease recurrence and to correlate the gene expression profile. In one such study, an 11-gene signature was identified to be associated with PCa recurrence and poor survival. Amongst them, a specific deubiquitinase called ubiquitin-specific peptidase 22 (USP22) was selectively and progressively overexpressed with PCa progression. Subsequently, it was shown to regulate androgen receptors and Myc, the two most important regulators of PCa progression. Furthermore, USP22 has been shown to be associated with the development of therapy resistant PCa. Inhibiting USP22 was also found to be therapeutically advantageous, especially in clinically challenging and advanced PCa. This review provides an update of USP22 related functions and challenges associated with PCa research and explains why targeting this axis is beneficial for PCa relapse cases.

## INTRODUCTION

Epidemiologically, prostate cancer (PCa) is the most common cancer in men and second most common cancer related death worldwide^[1]^. Over the past few years, treatment modalities have improved, albeit modestly, the overall survival of PCa patients. The fate of advanced PCa remains the same however, and androgen deprivation therapy (ADT) is the standard of care in such cases. PCa eventually recurs within 6 months to 2 years in the form of highly aggressive castration resistant prostate cancer (CRPC)^[2-5]^. While the treatment of CRPC with second generation ADT such as Abiraterone or Enzalutamide is promising, eventually, the cancer progresses to metastatic disease [called metastatic CRPC (mCRPC)], especially in the bone^[6]^. Chemotherapy with docetaxel is the first choice for treatment with mCRPC. Unfortunately, mCRPC patients have died due to complications related to metastatic transformation of PCa^[7-11]^. Interestingly, these mCRPC often lose the androgen-receptor dependency and are associated with the loss of tumor suppressor proteins such as tumor protein p53 (TP53) and retinoblastoma 1 (RB1)^[12,13]^. Genome-wide sequencing analysis has found some of the unique variations in the chromosomal sequence but no such driver mutation/s in PCa has been ascertained to correlate with cancer progression^[14-16]^. Moreover, not all primary PCa cases progress to CRPC. Therefore, to understand the indolent vs. aggressive nature of PCa, gene-expression analysis is highly important. To correlate CRPC progression and to stratify the therapeutic regimen, high throughput sequencing analysis of various stages of PCa to correlate genetic expression profiles with the therapy-resistant state has been attempted. The expression of a 11-gene signature in primary prostate tumors was shown to correlate with therapeutic failure in PCa patients^[17]^. Further characterization has shown that this 11-gene signature is a powerful predictor of distant metastasis and poor survival. Amongst these eleven genes, a specific deubiquitinases, named USP22, has been overexpressed following PCa progression. Further evidence indicates the importance of USP22 in a multi-faced pathway, which often correlates with a poor prognosis of PCa independently^[18-20]^.

## CLASSIFICATION OF DEUBIQUITINATING ENZYMES

The protein ubiquitin (Ub) plays an important role in tissue homeostasis. Ub modification is a reversible phenomenon that is coordinated by the deubiquitination pathway^[21-24]^. Deubiquitinating enzymes (DUBs) belong to either cysteine proteases [such as ubiquitin-specific proteases (USPs), ubiquitin C-terminal hydrolases (UCHs), *etc*.] or metalloproteases [Jab1/Mov34/Mpr1 (JAMM)], which are important for maintaining normal physiological homeostasis^[21,25]^. Approximately 100 DUBs are encoded in the human genome. DUBs are involved in various physiological processes including the processing of Ub precursors, reversal of ubiquitination and removal of poly-ubiquitin chains^[20,26]^. Therefore, DUBs regulate a series of cellular processes and functions including proteolysis, apoptosis, cell cycle progression, gene expression, DNA repair, maintenance of telomeric length, spermatogenesis, and so on^[27-30]^. One such conserved ubiquitin-specific protease is USP22 and it has been well characterized, relating to various physiological and pathological processes^[19,31]^.

## UBIQUITIN-SPECIFIC PEPTIDASE 22

The ubiquitin-specific peptidase 22 (USP22) belongs to the USPs family of DUBs and is highly conserved from yeast to vertebrates. In yeast, the USP22 homologue known as Ubp8, is complexed with Sgf73, Sgf11 and Sus1 to form the deubiquitylase module (DUBm) of the SAGA (Spt-Ada-Gcn5 Acetyl transferase) complex. The SAGA complex has a multi-disciplinary role in gene-expression and RNA-transport. Like yeast Ubp8, USP22 also forms a DUBm complex with the human orthologue ATXN7L3, ENY2 and ATXN7 and functions as a DUB unit of the human SAGA complex^[32,33]^.

## ROLE OF USP22 IN CELLULAR PROCESSES

As a part of the SAGA complex, transcriptional activation by deubiquitination of lysine-123 of histone-H2B is enhanced^[34,35]^. Later it was identified that histone-H2A ubiquitination can be processed by USP22. Ubiquitination of H2A by the polycomb group of proteins is related to transcriptional repression. However, whether deubiquitination of H2Aub (monoubiquitinated histone) by USP22 reverses the phenotype is not yet clearly established^[36]^.

Other than histones, USP22 also regulates the ubiquitination status of a large number of non-histone proteins. One of the most important functions of USP22 is to regulate telomeric length. Telomeric repeat binding factor 1 (TRF1) functions as a negative regulator of telomere length by inhibiting the access of telomerase to the telomeric region of chromosomes. Poly-ADP-ribosylation of TRF1 by Fbx4 releases it from the telomere, which in turn gets ubiquitinated and degraded by the proteasomal pathway. On the contrary, as a part of the SAGA complex, USP22, by deubiqitinating TRF1, restores its protein level and thereby maintains telomeric length. Depleting USP22 decreases TRF1 levels and enhances cell death by genotoxic insults^[27]^. The deubiquitination activity of USP22 is also important for the stability of Sirtuin 1 (SIRT1)^[37]^. By deacetylating, SIRT1 negatively regulates the transcriptional activity of p53 and thereby, p53 dependent apoptosis^[38]^. Deubiquitination also stabilizes another important transcription factor called c-Myc by the similar SIRT1 mediated pathway. In short, by deubiquitinating a number of transcriptional regulators such as Hes1, NFAT, COX-2, SNF1, *etc*., USP22 maintains their homeostatic functions within the cell^[39-42]^. A compensatory mechanism also involves SAGA, c-Myc and SIRT1. The enhanced stability of c-Myc through a USP22 dependent manner increases its transcriptional activity, which in turn, increases SIRT1 expression. However, the increase in SIRT1 levels enhances its deacetylation activity, which in turn, deacetylates USP22 and other SAGA components, thereby decreasing the enzymatic activity of USP22^[43]^. Interestingly, not all deubiquitinations altered protein stability; it also changes the molecular function of the protein. Deubiquitinating lysine-63 of FBP1 enhances its recruitment to the chromosome^[44]^. The role of USP22 in B cells is also important for its functionality. Complete ablation of USP22 in primary B cells was found to impair the classical non-homologous end joining and thereby, affects both V(D)J recombination and class switch recombination for the development of various IgG and IgE subtypes^[45]^.

Little is known about USP22 regulation however. Reports indicate that USP22 transcription is regulated by Sp1 and the PKA/CREB dependent pathway^[46,47]^. USP22 is also regulated and stabilized by phosphorylation. Phosphorylation of USP22 at T147 and S237 by CDK1 increases the deubiquitination status of cyclin B1 in a cell cycle dependent manner. USP22 mediated deubiquitination of Cyclin B1 promotes its nuclear accumulation and cell cycle progression^[48]^. USP22 is ubiquitously expressed in human subjects as well as in mice. In mice, USP22 expression was detected as early as in E4.5. Loss of both USP22 alleles results in an embryonic lethality starting at E10.5 and no live embryos were recovered after E14.5. Embryonic expression patterns indicate that the potential functions of USP22 relate to the development of extra-embryonic tissues and the loss of function of embryonic USP22 fails to establish vascular interactions with the maternal circulatory system, which leads to immense hypoxic stress induced lethality. Loss of USP22 is also associated with impairments in transforming growth factor β, vascular endothelial growth factor receptor-2 and platelet derived growth factor signaling axes in endothelial cells, and pericytes have been shown to be implicated with detrimental effects on cell survival, differentiation and vessel formation. However, the heterozygous loss of USP22 in mice is still viable but with retardation of growth and brain development^[38,45]^. USP22 expression is also important for embryonic stem cell (ESC) differentiation into the embryonic body where Sox2 expression needs to be suppressed. Studies have reported that USP22 functions as a transcriptional repressor by occupying and deubiquitinating H2B at the Sox2-promoter region during the differentiation of ESC into the embryonic body. USP22 expression is also important for regulating neural stem/progenitor cell maintenance through the Notch signaling pathway^[18]^. Deubiquitination by USP22 stabilizes the expression of Hes1 protein that negatively modulates neuronal differentiation. On the contrary, depletion of USP22 delays Hes1 oscillation and thereby, induces neuronal differentiation from neuronal progenitor stem cells^[39]^. Overall, USP22 functions in multiple pathways to maintain cellular homeostasis and physiological functions of cells.

## USP22 EXPRESSION IS FREQUENTLY ALTERED IN CANCER

Altered expression of USP22 was first detected in microarray studies from patient tissue cohorts where the expression of 11-gene signatures in stem like cells correlates with poor prognosis of the cancer^[17]^. Over the years, upregulation of USP22 has been validated in several cancers such as breast, colorectal, pancreatic, lung, ovarian, bladder, lymphoma, glioma, mesothelioma, neuroblastoma, *etc*.^[17,31,49-53]^. USP22 mainly functions as a part of the SAGA complex and depletion of USP22 alters the expression of a variety of transcriptional regulators that ultimately affect the cellular conserved pathway or cell metabolism^[54]^. On the contrary, overexpression of USP22 often stabilizes the transcriptional effector proteins that directly or indirectly influence gene expression. Higher expression of USP22 is associated with increased risk of cancer recurrence and poor disease-free survival^[19]^. USP22 expression also correlates with cell cycle progression. In fact, depletion of USP22 has been shown to be associated with cell cycle arrest, mainly at the G0/G1 phase^[18,48,55,56]^. Moreover, the depletion of USP22 was shown to decrease *in vivo* tumor growth^[19,55]^.

The oncogenic role of USP22 in cancer stem cells (CSC) has been identified as a poor prognostic factor in multiple cancer models. Mechanistic studies indicate that by deubiquitination, USP22 has been associated with the stabilization of a variety of its downstream proteins that are important for the development and maintenance of CSC including BMI1. It has also been shown that the increased stability of BMI1 induces CSC populations by inducing the expression of stemness associated genes such as CD133, SOX2, OCT4 and NANOG and thereby, favor the progression of gastric cancer^[57]^. The role of USP22 and BMI1 in glioma associated stem cells has also been reported. Under hypoxic conditions, USP22 stabilizes BMI1 to induce CSC formation for cancer progression in glioma models^[58]^.

In non-small cell lung cancer (NSCLC), the upregulation of USP22 was reported to be associated with advanced stage or recurrent NSCLC and considered as a poor prognostic marker for overall survival^[59]^. Knockdown of USP22 in an *in vivo* model was shown to decrease tumor angiogenesis, impair non-homologous DNA damage repair pathways and significantly improve the therapeutic efficacy of cisplatin. USP22 upregulation affects a broad range of pathways in NSCLC to maintain tumor aggressiveness. Cisplatin-resistant lung adenocarcinoma cells were shown to be associated with upregulation of USP22. According to that model, USP22 enhances DNA damage repair and cisplatin resistance by deubiquitinating histone H2A, which in turn facilitates the phosphorylation of histone H2AX. In addition, USP22 was shown to decrease the acetylation of Ku70 by stabilizing SIRT11 via deubiquitination. Ku70 acetylation dissociates the Bax-Ku70 interaction and thereby, induces apoptosis by favoring mitochondrial translocation of Bax. However, upregulation of USP22 in lung adenocarcinoma inhibits Bax-mediated apoptosis in cisplatin-resistant cells^[52]^. Upregulation of USP22 was also shown to be associated with chemotherapy-resistant pancreatic cancer cell survival by enhancing autophagic activity^[60]^. In breast and colorectal cancer, upregulation of USP22 was reported to be associated with decreased therapeutic efficacy of the HSP90 inhibitor ganetespib. Depletion of USP22 in an *in vivo* model of colorectal cancer was shown to increase the therapeutic potentiation of ganetespib^[61]^.

In gastric cancer, the co-expression of USP22 and BMI1 was shown to be associated with shorter disease-free survival and a poor prognosis for overall survival^[62]^. This was similarly reported in colon cancers. The upregulated expression of USP22 was significantly correlated with both a decrease in relapse-free survival and overall survival. An *in vitro* study showed that the upregulation of USP22 mediated the enhanced expression of BMI1 and Cyclin D2, and was responsible for increased cell proliferation and the metastatic behavior of colon cancer cells^[63]^. In hepatocellular carcinoma, the enhanced expression of USP22 was shown to be an independent factor for a poor prognosis with tumor progression^[64]^. The enhanced stability of c-Myc following USP22 mediated deubiquitination was reported to be associated with breast cancer cell proliferation and metastatic activity^[43]^. The upregulation of USP22 was also reported to be associated with a poor prognosis in papillary thyroid carcinoma^[65]^ and glioma^[66]^. In retinoblastoma, the depletion of USP22 has been shown to induce cancer cell apoptosis by suppressing the TERT/P53 signal pathway^[67]^.

In the majority of cancers, USP22 functions like an oncogene. Tumor suppressive functions however, were also reported in certain cancer models such as acute myeloid leukemia (AML) and colorectal cancer. Recently, in an *in vivo* model, it was shown that the deletion of USP22 from *Mx1-Cre* mice carrying *Kras^G12D/+^* was associated with shorter survival compared to *Kras^G12D/+^* mice. Further studies indicate that mice that received myeloid progenitor cells carrying USP22 deletion and mutated *Kras^G12D/+^* had an AML phenotype. As a mechanism, USP22 was shown to positively regulate protein expression of the transcription factor PU.1, which is important for myeloid and B-lymphoid cell development. Depletion of USP22 directly affected myeloid specific gene expression in *Kras^G12D/+^* mutated mice, which further led to the development of AML^[68]^. Contradictory functions of USP22 in the development of colorectal cancer have been reported. One such study showed that intestine specific *USP22* deletion impaired the tumor phenotype associated with *Apc* mutation and positively correlated with the intestinal tumor burden and decreased survival. Mechanistically, the loss of USP22 resulted in increased mTOR activity and has been linked to the tumorigenic properties of colorectal carcinoma^[69]^.

Over-expression of USP22 is observed in aggressive PCa and has been associated with its oncogenic function. In the following section, we will concentrate mainly on the role of USP22 in the development of CRPC and treatment-resistant PCa.

## USP22 AND PROSTATE CANCER

**Figure 1.**
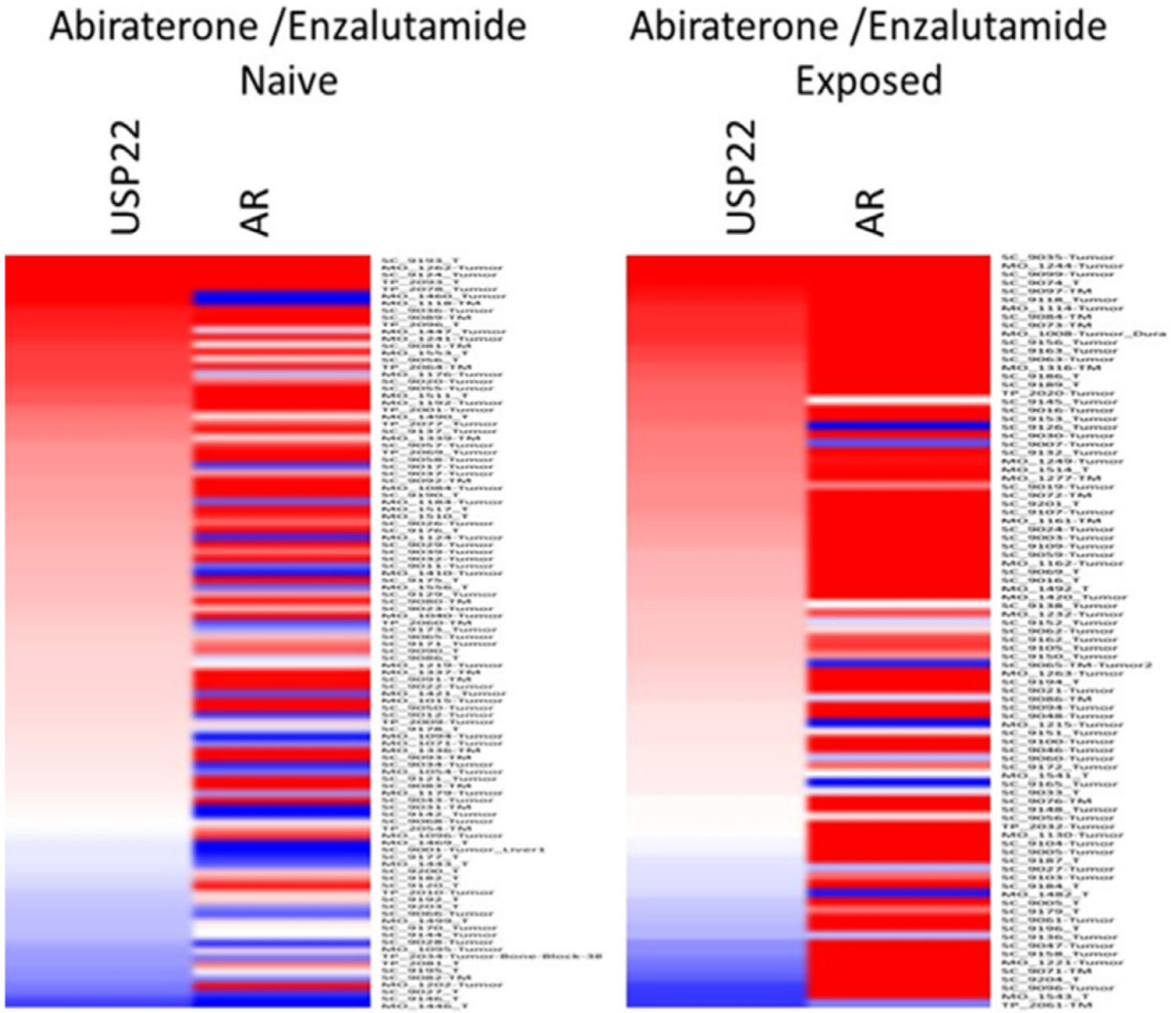
Analysis of ubiquitin-specific peptidase 22 (USP22) and androgen receptor (AR) expression from metastatic biopsy samples deposited in https://github.com/cBioPortal/datahub/tree/master/public/prad_su2c_2019

**Figure 2.**
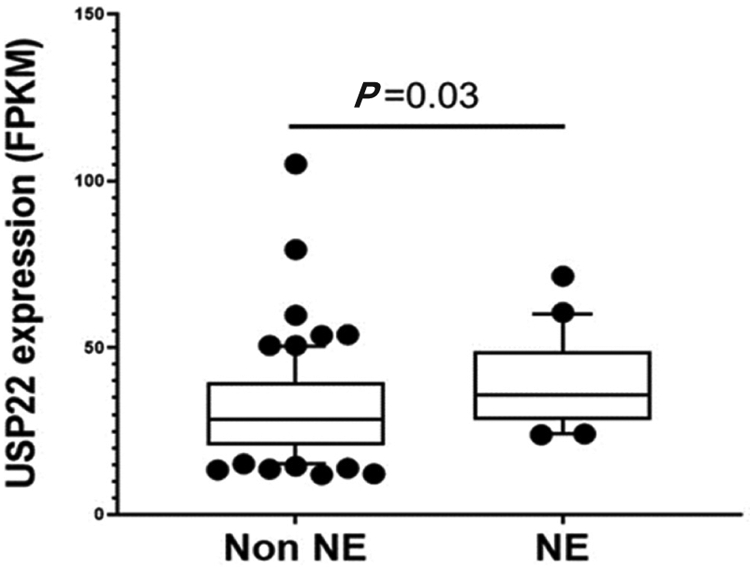
Ubiquitin-specific peptidase 22 (USP22) expression between neuroendocrine (NE) *vs*. patients who did not develop neuroendocrine PCa (Non NE) using the GSE126078 database

**Figure 3.**
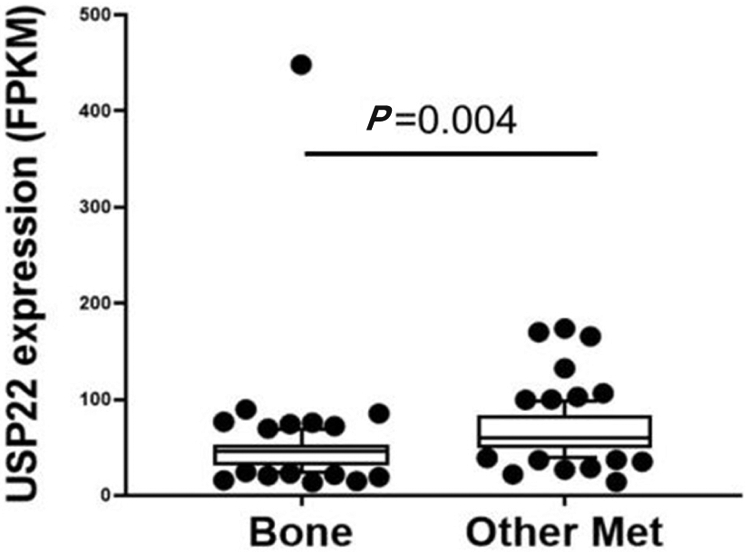
Ubiquitin-specific peptidase 22 (USP22) expression compared between bone and visceral metastatic sites (Other met) using the expression data deposited in cbioportal (https://www.cbioportal.org/study/summary?id=prad_su2c_2019, SU2C/PCF Dream Team, PNAS 2019)

During PCa progression, increase in copy numbers as well as enhanced expression of androgen receptor (AR) (along with its splice variant formation) often led to aggressive therapy resistant phenotypes^[70,71]^. Therefore, targeting AR is the most favorable choice to limit PCa progression. Over the years, improvement in AR targeted therapy has increased overall survival to some extent, however, recent clinical studies indicate that a individuals are becoming resistant to second generation anti-androgen therapy. Therefore, understanding therapy resistance pathways may provide better or alternative solutions to target PCa. Since the 11-gene signature was shown to predict PCa recurrence and therapy resistance, the contribution of individual genes and 5-year PCa survival was analyzed in mCRPC cases. High expression of Ki-67, BUB1, KNTC1 and USP22 showed significant association with poor 5-year survival^[18]^. Further, it was shown that the concerted expression of USP22, AR and Myc in PCa cells predicted the worst prognosis of the disease. USP22 plays an important role in AR protein stability and recruitment to AR-binding regions to drive AR driven cancer cell proliferation and tumor growth in CRPC cells. Later, it was shown that USP22 is equally important for protein stability of AR-variants. The upregulation of USP22 also promotes AR/Myc driven gene expression independent of androgens in the CRPC cell line, implicating that USP22 has a tremendous impact on genes that are regulated by AR and Myc in CRPC cells. This might be important to the phenomenon of anti-androgen therapy resistance^[18]^. Interestingly, analysis of patient data with mCRPC validates that point (https://www.cbioportal.org/)^[72]^. Analysis of the coordinated expression of USP22 and AR between abiraterone/enzalutamide (2nd generation anti-androgen therapy) in naïve vs. exposed groups revealed that USP22/AR expression is upregulated in patients who have progressed to mCRPC under treatment conditions. However, in the treatment naïve group, such correlation was not ascertained [Figure 1]. Patients who are resistant to abiraterone/enzalutamide therapy often develop neuroendocrine-like PCa. Further analysis of patient data (GSE126078) indicates that in pathologically validated neuroendocrine PCa, USP22 expression is significantly higher compared to metastatic sites, which did not develop the neuroendocrine phenotype [Figure 2]. In general, bone is the preferred metastatic site for PCa. However, neuroendocrine PCas often develop visceral metastasis. USP22 expression in visceral metastatic sites (https://www.cbioportal.org/)^[72]^ was significantly higher compared to bone [Figure 3]. Therefore, further validation of the earlier observations and selective upregulation of USP22 are associated with therapy resistance and progression of the disease. This group of patients need an alternative form of treatment and the early detection and stratification of these patients will be beneficial.

**Figure 4.**
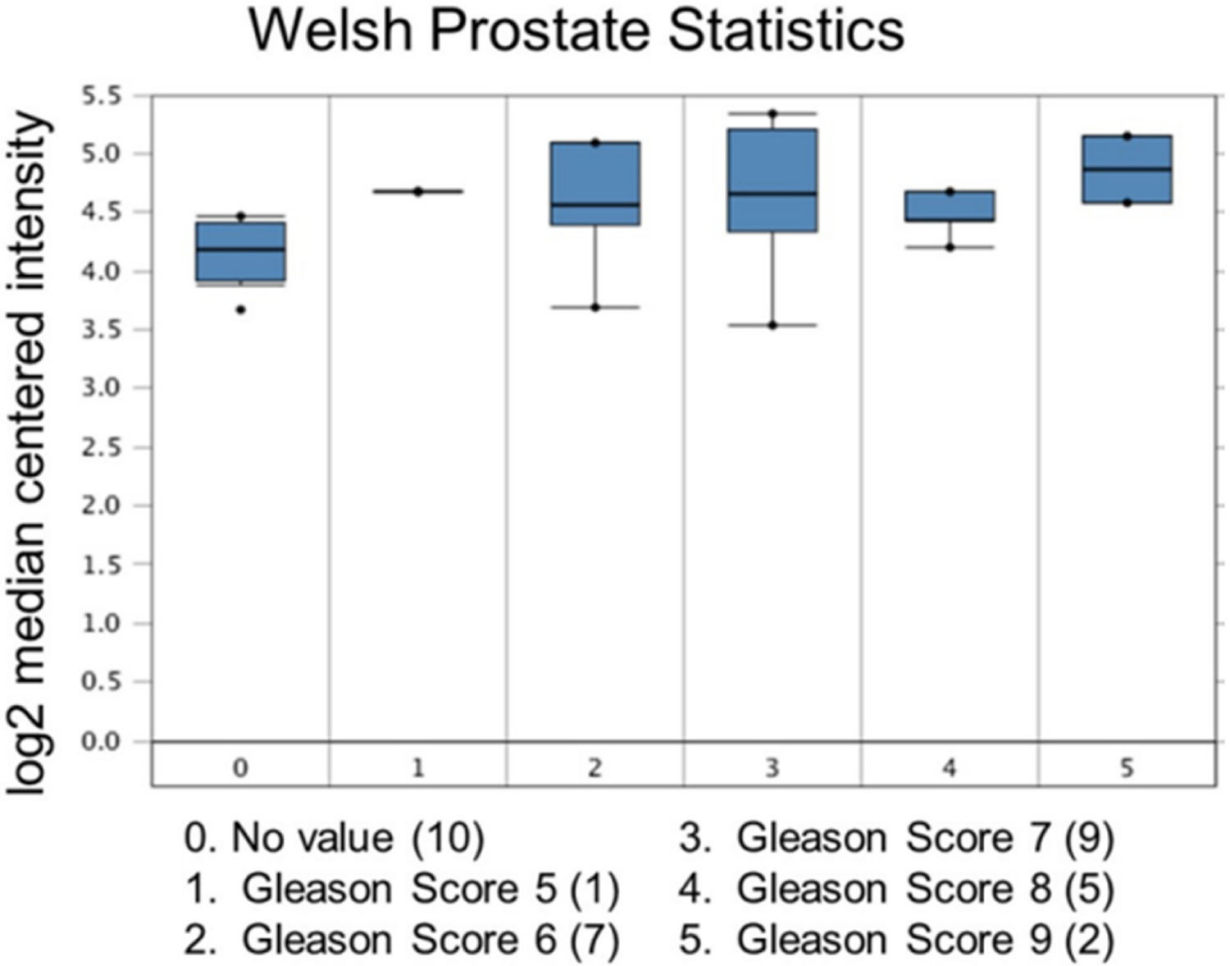
Ubiquitin-specific peptidase 22 expression across the Gleason Score. Number of patients are in parenthesis

**Figure 5.**
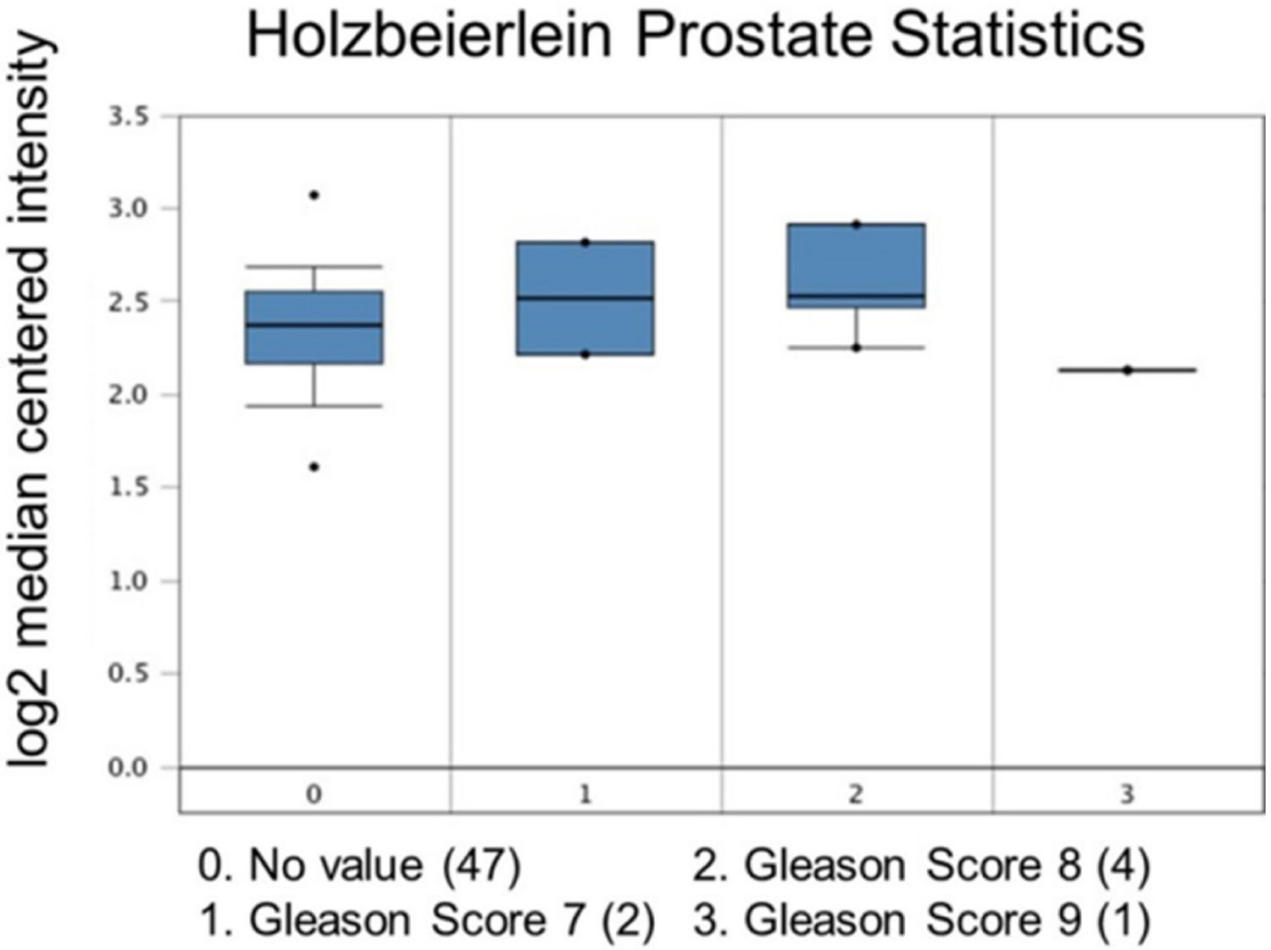
Ubiquitin-specific peptidase 22 expression across the Gleason Score. Number of patients are in parenthesis

**Figure 6.**
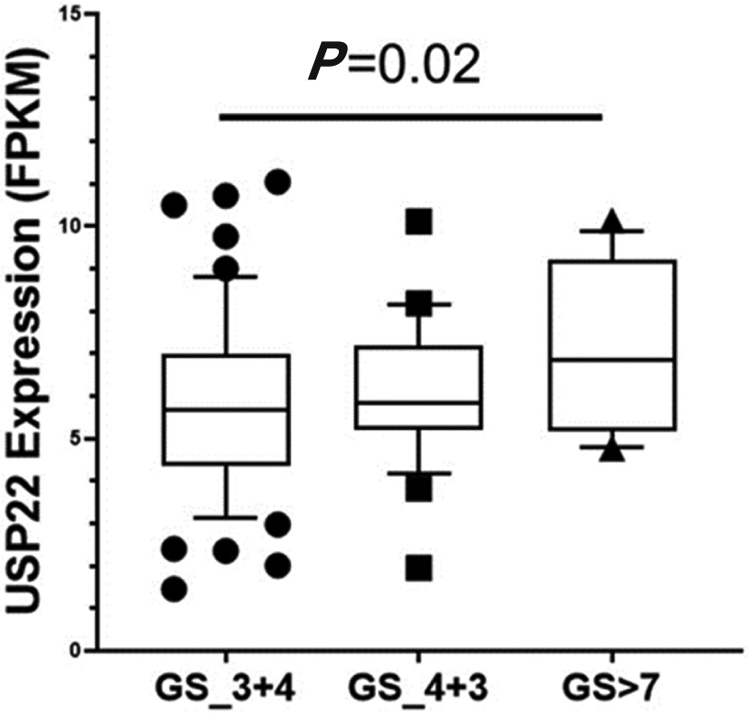
Ubiquitin-specific peptidase 22 expression across the Gleason Score (GS) using the database GSE54460

**Figure 7.**
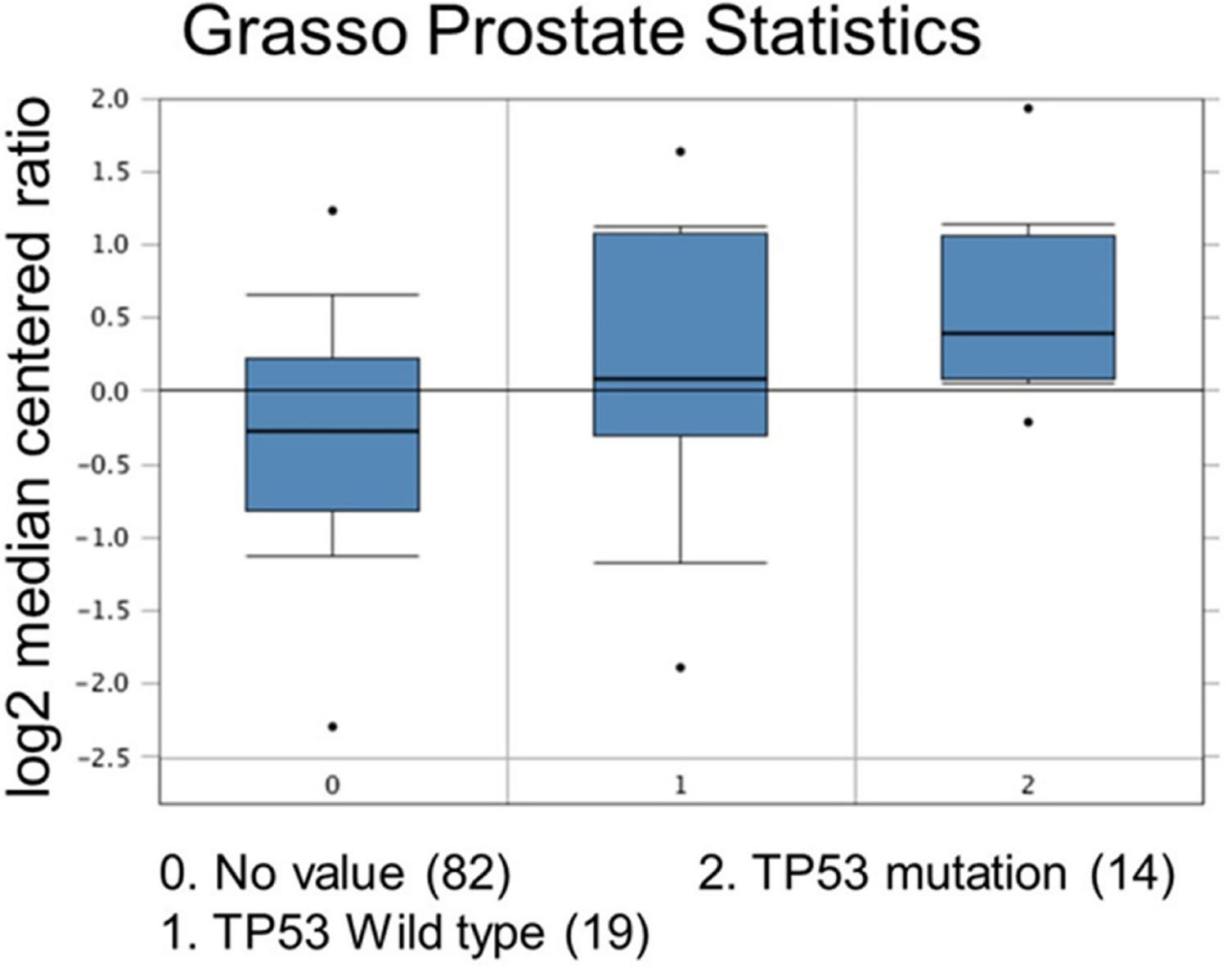
Comparison study for ubiquitin-specific peptidase 22 expression with TP53 mutation status. Number of patients are in parenthesis

Increased expression of USP22 was also observed with progression of primary PCa. Analysis of the Oncomine database showed that USP22 expression increases with increased Gleason score [Figures 4 and 5]^[73,74]^, indicating that during progression of PCa, USP22 expression can be a predictive factor for advanced disease. Further analysis of GSE54460 expression data supports increased USP22 expression with higher grades of PCa [Figure 6]. Moreover, advanced PCa patients often have functionally inactive TP53. Oncomine analysis of the Grasso cohort indicates that TP53 mutation is associated with increased expression of USP22 [Figure 7]^[75]^. Therefore, primary PCa patients who have higher expression of USP22 with functionally inactive TP53 might be candidates for alternative therapeutic approaches. Various clinical trials are currently ongoing with upfront administration of chemotherapy such as cabazitaxel for patients who have started to develop disease progression in the early stage. In future, analysis of such cohorts will address whether such therapy can be beneficial for those who have showed early upregulation of USP22 expression.

To mimic that hyperactivation/overexpression state, the role of USP22 functions in PCa progression has recently been redefined in a genetically modified mouse model. According to the model, prostate specific upregulation of USP22 is associated with a hyperproliferative phenotype, an indication of aberrant cell proliferation. Moreover, studies have also showed that overexpression of USP22 is important for cellular survival following a genotoxic insult by DNA-damaging agents. In line with their finding, the authors have identified the nucleotide excision repair pathway protein XPC as a substrate for USP22, which modulate XPC polyubiquitination status following the DNA-damage response and thereby, efficiently recruited it into the damage foci. Interestingly, the depletion of USP22 in PCa cells affects efficient DNA repair and therefore, presents a therapeutic challenge^[19]^.

Although USP22 was identified almost 15 years earlier as an important oncogenic driver for therapy resistant prostate cancer, not much work has been carried out to understand its importance in the development of mCRPC. As part of the SAGA complex, how inappropriate stoichiometric upregulation of USP22 in PCa drives AR/Myc mediated gene-expression remains unresolved. Also, whether USP22 plays an independent role in PCa progression is not well understood.

## OTHER USPS IN PROSTATE CANCERS

Importantly, other ubiquitin specific proteases or USPs have long been recognized in the progression of advanced PCa.

USP2a (also known as USP2) has been associated with PCa development. More than 50% of cases with PCa have USP2a overexpression. Increase in USP2a selectively deubiquitinates and stabilizes MDM2, which is important for the proteasomal degradation of p53 in PCa cells. p53 is the negative regulator of Myc in many cases. USP2a mediated enhanced stability of MDM2 abrogates p53 accumulation and its tumor suppressive functions. Therefore, the inhibition of p53 mediated transactivation of transcriptional activity indirectly stabilizes Myc accumulation in cells and thereby, enhances the development of aggressive PCa transformation. The deubiquitination activity of USP2a was also found to stabilize the anti-apoptotic gene fatty acid synthase and thereby induce cells to develop neoplastic transformation. The depletion of USP2a has also been shown to abrogate such cellular transformation^[76,77]^.

USP7 has been associated with PCa and plays a negative role for PTEN nuclear localization. PTEN is generally regarded as a protein phosphatase that dephosphorylates the phosphatidylinositol (3,4,5)-triphosphate to inhibit AKT signaling. However, PTEN’s role in the nuclear DNA repair system associated with tumor suppressive functions has been well recognized. Following mono-ubiquitination, PTEN moves into the nucleus and participates in the repair processes. In PCa, over-expression of USP7 expels this ubiquitinated-PTEN to the cytosol and activates the cells towards transformation. Interestingly, in the presence of androgen, USP7 was identified as a co-regulator of AR. Studies also suggest that USP7 mediated AR-deubiquitination enhance the AR-transcriptional ability that promotes cell proliferation and PCa aggressiveness. Moreover, single nucleotide polymorphisms that affect USP7 function has been associated with the development of intermediate risk PCas^[78,79]^.

USP19 silencing directly affects the growth of several prostate cancer cell lines, suggesting a putative role in carcinogenesis^[80]^. USP19 deubiquitinates and stabilizes KPC1, an E3 ligase for p27. Interestingly, the effects of decreased nuclear levels of p27, resulting in a poor prognosis, have already been described in prostate cancer^[81]^. USP19 regulates the levels of p27, although p27 is not a USP19 substrate. Reports indicate that the disruption of USP19 inhibits a series of PCa cell proliferation by arresting cells in the G1 to S phase transition through stabilization of the cyclin-dependent kinase inhibitor p27^[80]^. Increased stability of AR by USP12, USP14 and USP26 has been linked to the development of aggressive PCa^[82-84]^. Recent reports indicate that the overexpression of USP33 in PCa confers docetaxel resistance by inhibiting JNK activation and apoptosis^[85]^.

In the context of PCa, most USPs are overexpressed; however, USP9x was found to be down regulated in advanced PCa and was associated with higher Gleason scores. This downregulation increases the local invasiveness of PCa cells, possibly through the ERK activation pathway^[86]^.

Among all the DUBs, available data suggest that USP22 functions often overlapped with other reported USPs in the context of progression and development of therapy resistant PCas. Therefore, USP22 targeted therapy or broad-spectrum inhibitors that can abrogate the functions of a group of USPs may be a better therapeutic agent in PCa.

## TARGETING USP22 IN ADVANCED PROSTATE CANCER TREATMENT

Recent studies have suggested that USP22 is emerging as a potential oncogenic driver in relation to PCa. As a member of the cysteine protease family, its catalytic domains are somewhat conserved amongst family members. Therefore, the development of inhibitors specifically against one such member is challenging. Efforts have been made to develop small molecule inhibitors against the allosteric sites of USP22. However, till now, no such specific inhibitor has been validated to target USP22. Recently, Pirarubicin (4’-O-tetrahydropyranyl doxorubicin, THP), an anthracycline (analogue of another chemotherapeutic agent known as doxorubicin), has been shown to inhibit USP22 expression in a condition-specific manner^[87]^. Reports indicated that protein kinase A (PKA), protein kinase B or mitogen activated kinase-mediated phosphorylation of CREB-1 bind and activate the USP22 promoter for its synthesis. The addition of THP abrogates PKA activity and decreases CREB-1 phosphorylation,, thereby inhibiting USP22 expression and USP22 mediated tumorigenic activity. Betulinic acid (BA), a small molecule isolated from white birch trees, has been shown to inhibit an array of DUBs. BA was also showed to reduce AR protein stability and selectively kills PCa cells. Another multi-DUB inhibitor WP1130 has been shown to selectively kill PCa cells. Treatment with WP1130 also reduces AR expression in CRPC cells. Therefore, BA and WP1130 have the potential to enhance the therapeutic efficacy of CRPC cells and the published literature suggests that the combination of these inhibitors with enzalutamide increases the therapeutic window for the treatment of advanced PCa patients^[88]^. With such growing knowledge, scientists have tried to develop exosite inhibitors against the various DUBs. One such inhibitor, P5091, is highly selective against USP7 and has been shown to induce apoptotic cell death in therapy resistant multiple myeloma cells^[89]^. However, its selectivity and specificity as an agent in PCa remains unknown.

## CONCLUSION

**Figure 8.**
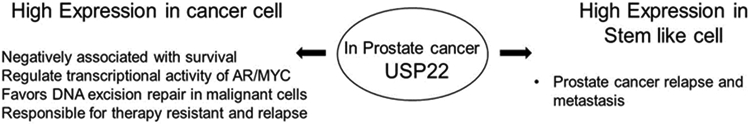
Summary of ubiquitin-specific peptidase 22 (USP22) role in prostate cancer

In summary, the oncogenic role of upregulated USP22 in the progression and development of treatment resistance of PCas has been observed [Figure 8]^[19]^. Accumulated evidence indicates that USP22 possibly functions independent of the SAGA complex in the progression of PCas. Increased acetylation and enhanced activity of GCN5 has been reported to be associated with advanced PCa. However, there is a lack of studies to ascertain any relationship between upregulated USP22 and other members of the SAGA complex in the development of aggressive PCas. Moreover, in advanced PCas, the coordinated function of upregulated Myc and USP22 indicates the lack of feedback regulation by hyperactivated Myc. Therefore, to develop better targeted therapeutic approaches, a comprehensive understanding about the functional interactions among the various sub-units of SAGA and their relationships with AR/Myc is important. Moreover, the differential functions of USP22 in the normal prostate, aggressive disease and disease progression are not fully understood. Thus, defining the role of USP22 will be beneficial for the development of future therapeutic modalities.
